# Toward Exploring and Utilizing the Nutritional and Functional Properties of Cereal Crops

**DOI:** 10.3390/foods12050976

**Published:** 2023-02-25

**Authors:** Yaqiong Wang, Min Tu, Guangyuan He, Yin Li, Junli Chang

**Affiliations:** 1 The Genetic Engineering International Cooperation Base of Chinese Ministry of Science and Technology, The Key Laboratory of Molecular Biophysics of Chinese Ministry of Education, College of Life Science and Technology, Huazhong University of Science & Technology, Wuhan 430074, China; 2 Hubei Technical Engineering Research Center for Chemical Utilization and Engineering Development of Agricultural and Byproduct Resources, School of Chemical and Environmental Engineering, Wuhan Polytechnic University, Wuhan 430023, China

## 1. Introduction

Cereal crops are of great importance in the development of human civilization and fall into two groups, major crops and minor crops. Wheat (*Triticum aestivum* L.), maize (*Zea mays* L.), and rice (*Oryza sativa* L.) belong to major cereal crops, as they are the top three most produced cereals in the world. The minor cereals usually include sorghum (*Sorghum bicolor* L.), rye (*Secale cereal* L.), oats (*Avena sativa* L.), foxtail millet (*Setaria italica* L.) and teff (*Eragrostis Tef*), as well as many other under-utilized cereals preferentially cultivated in certain countries or regions. Recently, increasing attentions have been drawn to the nutritional and functional properties of not only the major cereals but also those minor crops. Along with economic development, the demands from modern consumers for healthy diets with improved nutritional and functional properties have been greatly expanded. These consumers’ demands, in turn, impact on both basic and applied research related to major and minor cereals. Correspondingly, the emerging trend of “eat full” to “eat well” holds the potential to become “mainstream”.

Cereal grains are composed of three major components, starches, proteins, and lipids, with other minor albeit important ingredients, such as minerals, fibers, vitamins and bioactive compounds. Numerous bioactive compounds (for example, phenolic acids, flavonoids, tannins, carotenoids, and saponins) have been identified and characterized in different cereal seeds, especially those from the minor cereals. Studies have generally established that although the seeds of major cereals are rich in starch and storage proteins, they have less healthy grain ingredients (e.g., fibers, minerals, vitamins and bioactive compounds) compared to seeds from the minor cereals. Therefore, these minor cereals have re-gained the attentions of consumers, breeders and scientists owing to their excellent nutritional properties. Nutritional studies have recognized that increased consumption of healthy grain ingredients through the addition of these minor cereals (pseudocereals as well) into our daily diet has been associated with protection against diseases such as cancer, type II diabetes, obesity, cardiovascular diseases (CVDs) and celiac disease, or anemia.

In this background, the present Topic “Nutritional and Functional Properties of Cereal Crops” provides a forum for researchers to communicate their latest findings related to the nutritional and functional properties of cereal. We expect a diverse set of studies covering multi-disciplines, and therefore collaborate with several related journals, including *Foods*, *Antioxidants*, *Nutrients*, *Metabolites* and *Crops*. This topic have collected a cohort of diverse, high-quality articles with a relatively low acceptance rate (~26%), falling into two categories: (1) the “upstream” studies related to cereal nutritional and functional properties, for instance to compare the diversity of metabolites between different cereal grains, and to investigate the effects of technological steps on the nutritional compounds in the cereals; (2) the “downstream” studies, such as those evaluating the effects of healthy cereal ingredients on human diseases. This Editorial paper aims to showcase the eight papers (including two reviews and six original research papers) and discuss related perspectives.

## 2. “Upstream” Studies Related to the Nutritional and Functional Properties of Cereals

Tang et al. [1] compared flavonoid and carotenoid metabolites in the seeds of six Poaceae species (i.e., wheat, maize, rice, sorghum, foxtail millet, and broomcorn millet), identified a total of 201 flavonoids and 29 carotenoid metabolites with flavone, anthocyanins, flavanone and polyphenol being the major metabolic differences between the species.

Carcea et al. [2] performed comparative studies of the effects of stone milling and roller milling techniques on the nutritional indicators (e.g., phytic acid and trans-fatty acid contents, and starch damage) and revealed that starch damage, but not the other two indicators, was associated with the milling method.

With the recent advancements in modern analytical technologies and the recognition of agricultural sustainability, minor cereals (such as millets) or even pseudocereals (for example, amaranth and quinoa) have attracted increasing attentions because they are environmental-friendly and offer a plethora of nutritional benefits. These advantages and the recent exploration and application of the benefits have been reviewed by Balakrishinan et al. [3]. This comprehensive review covers the nutritional significance of these crops, their emerging food applications and a summary of the current clinical trial evidence on the consumption of amaranth, quinoa and millets.

As detailed examples for the application of minor crops, Aung et al. [4] explored the application of germinated wheat as a beneficial ingredient of plant-based beverages and evaluate the sensory properties and consumer acceptability.

In another example, Zhang et al. [5] demonstrated that the oil extraction method of field muskmelon seeds affects the physiochemical and functional properties of the extracted proteins as well, potentially influencing the applications of those proteins as food ingredients.

## 3. “Downstream” Studies Related to the Nutritional and Functional Properties of Cereals

This topic also collected several studies focused on the influences of cereal ingredients on health benefits. Sorghum is the world’s fifth largest cereal crop and in some sorghum varieties polyphenols are rich in the seeds with potential health benefits in the management of obesity and diabetes. Lee et al. [6] employed cell biology approaches and showed that the sorghum bran extracts with high phenolic contents, rather than those low in phenols, repress adipogenesis through repression of ROS production, MAPK signaling, and insulin signaling adipogenesis through repression of ROS production, MAPK signaling, and insulin signaling. These mechanistic insights justify the health benefits and potential use of highly phenolic sorghum varieties.

Another meta-analysis also focused on a cereal secondary metabolite, the oat β-glucan. Yu et al. [7] reviewed the randomized controlled trials of oat β-glucan interventions in hypercholesterolemia and concluded that oat β-glucan intake may significantly reduce the level of total cholesterol and low-density lipoprotein-cholesterol, supporting the health benefits of minor cereal-rich diets.

In contrast to the focus on secondary metabolism (polyphenols in sorghum), Huang et al. investigated the effects of different types of carbohydrates (i.e., simple, refined and unrefined carbohydrate-rich diets) on plasma metabolites by using a randomized and controlled cross-over trial, discovering that individual variation, but not the carbohydrate type, impacting more on the plasma metabolic profiles [8].

## 4. Concluding Remarks

Increasing medical trials are uncovering the health benefits of the consumption of minor cereal grains. However, the inter- and intra-species diversity of nutritional and functional properties has not been fully discovered in cereal crops. Advancements in multi-omic technologies offer powerful tools to fulfill such purposes, as exemplified by Tang et al. [1]. A combination of advanced techniques from multi-omics, plant science, food science and technology will further our knowledge of the nutritional and functional properties of cereals.

## Data Availability

Data is contained within the article.
